# Structure and electrochemical properties of copper wires with seamless 1D nanostructures

**DOI:** 10.1016/j.dib.2018.01.097

**Published:** 2018-02-03

**Authors:** Yutong Wu, Meiqi Gao, Song Li, Yuping Ren, Gaowu Qin

**Affiliations:** School of Materials Science and Engineering, Northeastern University, Shenyang 110819, China

## Abstract

A seamless Cu nanowire array grown on Cu wire is prepared by combining thermal oxidation method and electrochemical reduction. The data set described in this paper includes the structure of the Cu nanowires electrode, electrocatalytic active surface area, linear sweep voltammetry and amperometry measurement for nitrate sensing. The electrochemical data show that Cu nanowire arrays exhibited a linear response to nitrate ions over a concentration range from 50 μM to 600 μM (*R*^2^ = 0.9974) with a sensitivity of 0.357 μA μM^−1^ cm^−^^1^ and detection limit of 12.2 μM at a signal-to-noise ratio of 3, respectively.

**Specifications Table**

**Table:** 

Subject area	Materials science
More specific subject area	Sensing nanostructures
Type of data	Table, image, graph.
How data was acquired	X-ray diffraction (Rigaka X'Pert MPD system equipped with a Cu Kα X-ray source), SEM (FESEM, JSM-7001F, JEOL), electrochemical workstation (Zennium E, Zahner).
Data format	Raw, analyzed.
Experimental factors	Cu wires were sonicated in 1 M HCl solution for 3 min and then put into deionized water for 3 min before further treatment.
Experimental features	Sample preparation:
	✓ Cu wires were annealed in air at 600 °C for 4 h with a heating rate of 10 °C/min. ✓ Oxide CuO_x_ electrode was reduced at − 0.4 V (vs RHE) in N_2_ purged electrolyte. Electrochemical analysis of data: electrocatalytic active surface areas and detection abilities were obtained from electrochemical workstation with three-electrode system in prepared electrolyte.
Data source location	School of Materials Science and Engineering, Northeastern University, Shenyang 110819, China.
Data accessibility	Data is displayed within this article.

**Value of the data**

• Growth of nanowire arrays on Cu wires by combing thermal oxidation and electrochemical reduction.
• Using high-density seamless nanowire array grown on Cu wire as nitrate electrochemical sensor.
• Nitrate sensing properties of 1D nanostructured Cu wires.

## 1 Data

**Fig. 1:**
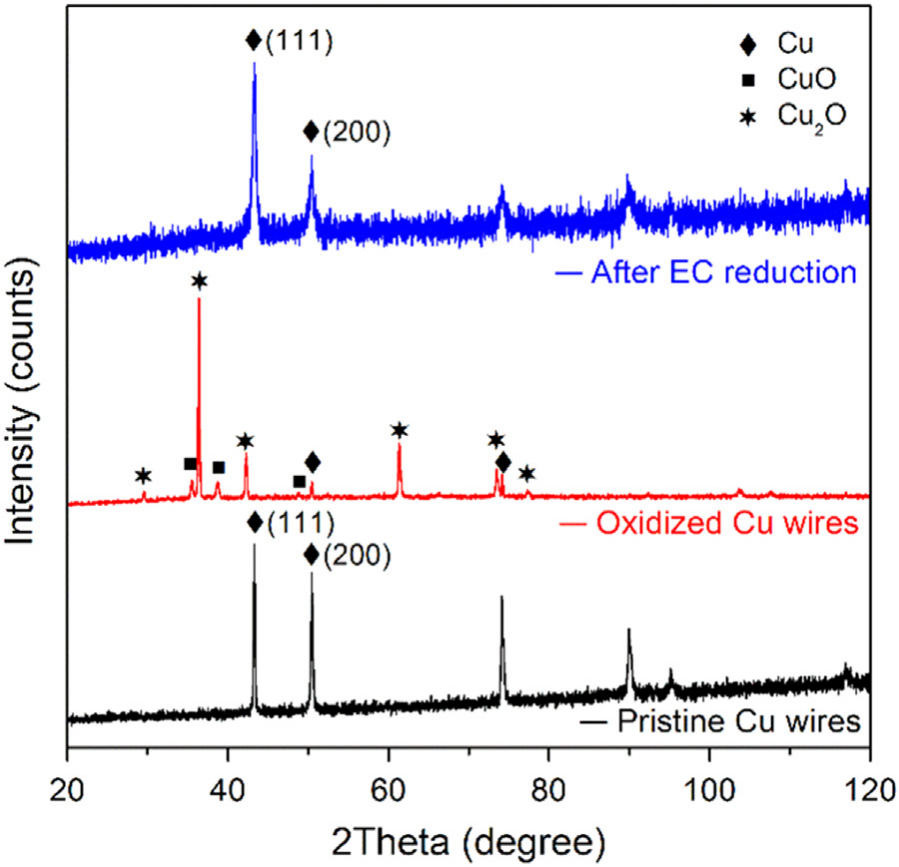
XRD patterns of Cu wire, Cu oxides nanowires and prepared Cu nanowires electrode.

**Fig. 2:**
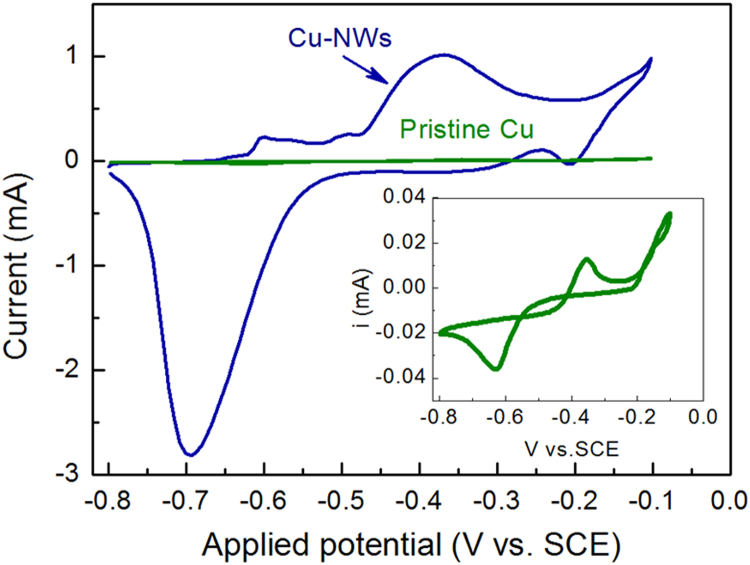
CV curves of Cu wires with and without nanowire structures in N_2_-purged 50 mM NaOH electrolyte at 5 mV s^−1^. Inset is the CV curve of pristine Cu wire electrode. Length of the wires: 5 cm.

**Fig. 3:**
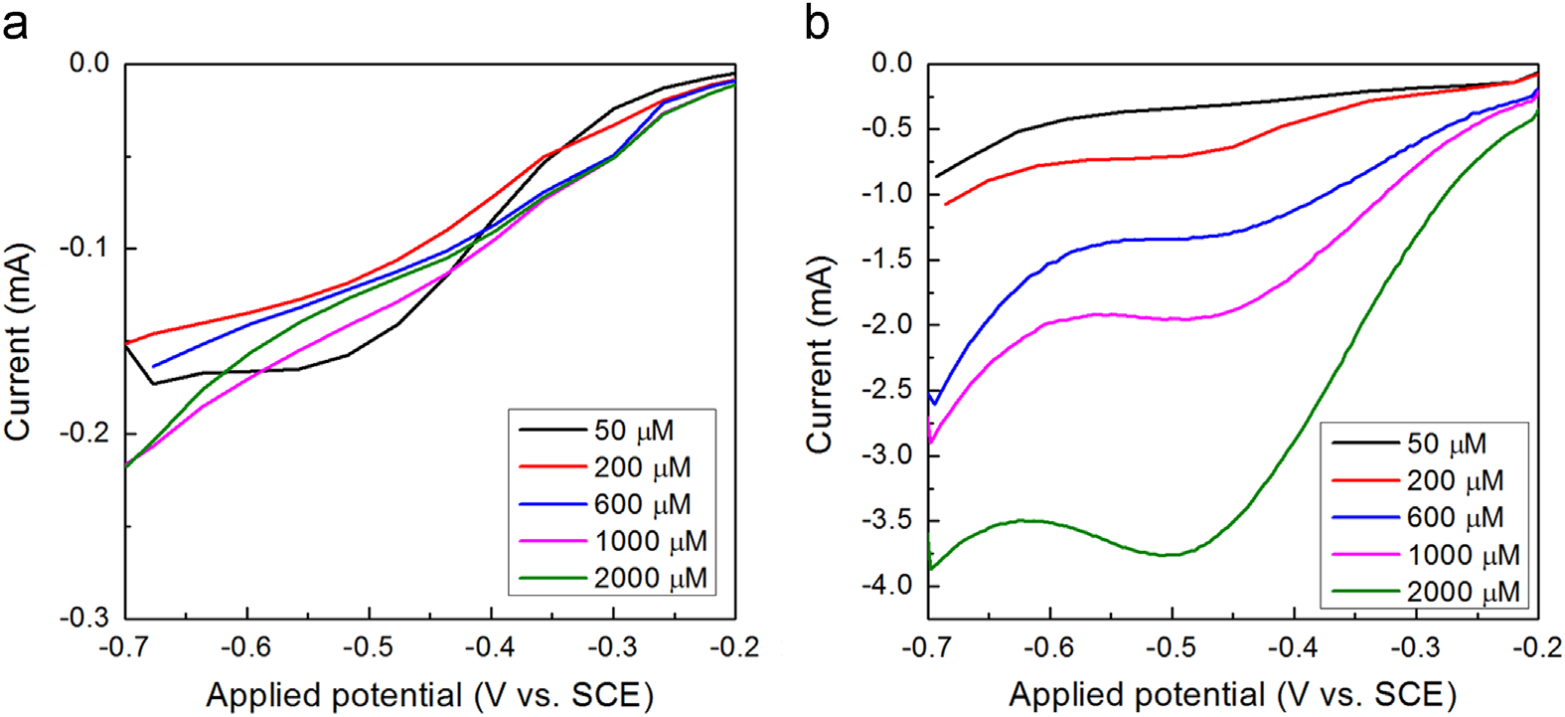
Typical LSV responses of (a) pristine Cu wire and (b) Cu-NWs electrodes in electrolytes with different nitrate concentration. Supporting electrolyte, 0.1 M Na_2_SO_4_ solution (pH = 2); scan rate, 40 mV s^-1^; length of the Cu wire, 15 cm.

**Fig. 4:**
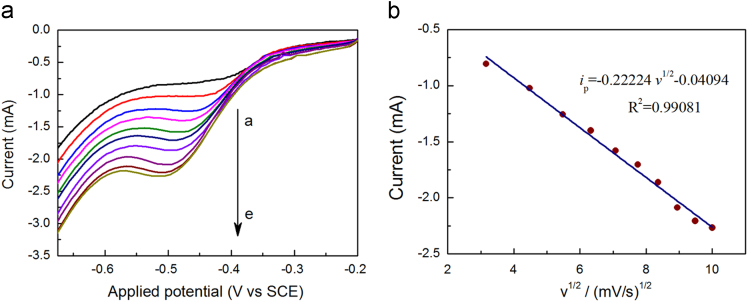
(a) LSV curves of Cu-NWs electrode of 0.1 M NaNO_3_ in 0.1 M Na_2_SO_4_ solution (pH = 2) by sweeping at different scan rates (from a to e, 0.01–0.1 V s^−^^1^ with step width 0.01 V s^−1^, respectively); (b) The linear dependence of peak current on the square root of the scan rate.

**Fig. 5:**
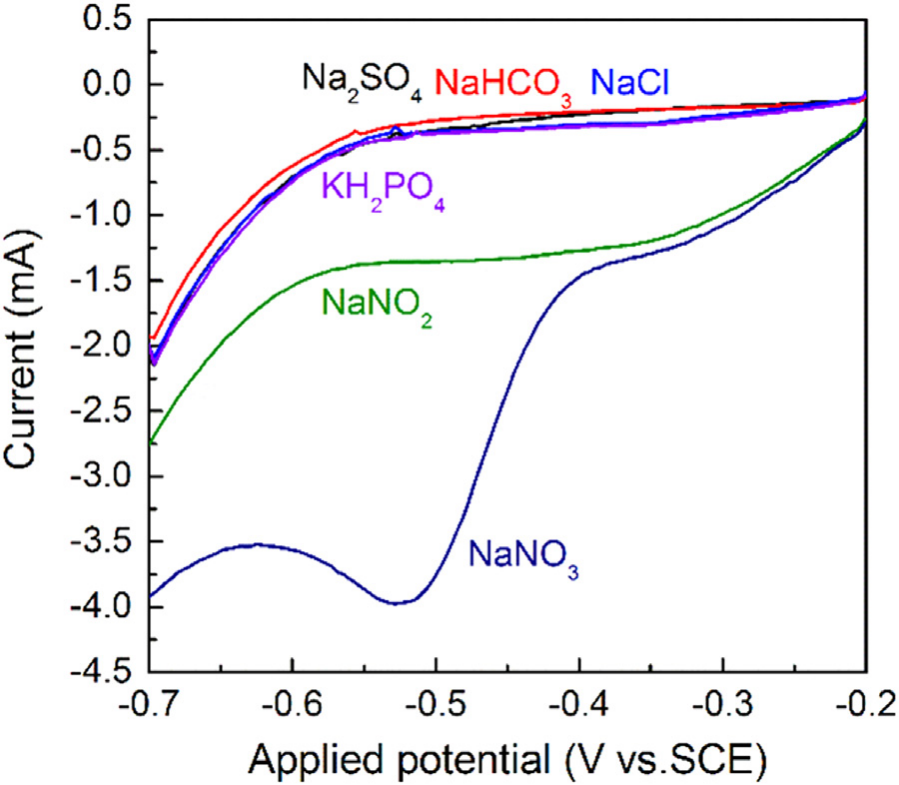
LSV curves of Cu-NWs electrode in 0.1 M Na_2_SO_4_ electrolyte (pH = 2) with sequential addition of 1 mM NaHCO_3_, 1 mM NaCl, 1 mM KH_2_PO_4_, 1 mM NaNO_2_, and 1 mM NaNO_3_.

The data set shows the crystal structure of Cu nanowires (Fig. 1), electrochemical active surface area (Fig. 2), Linear sweep voltammetry (LSV) responses of pristine Cu wire and Cu nanowires electrodes (Fig. 3, Fig. 4), anti-interference properties of Cu nanowires electrode (Fig. 5), comparison of the performances of various nitrate sensors (Table 1) and stability performance of the Cu nanowires electrochemical sensor (Table 2).

**Table 1:** Comparison of the performances of various nitrate sensors. *S*: sensitivity, *A*: area, DL: detection limit (signal/noise = 3), *R*^2^: Correlation coefficient.

Electrode materials	Analytical method	Linear range (μM)	*S*/*A*^a^ (μA μM^−1^ cm^−2^)	DL (μM)	*R*^2^	Ref.
Copper-plated copper wire	LSV	10–200	0.085	–	0.998	[2]
Cu sheet	Amperometry	100–250	0.0082	4.2	0.9991	[3]
Cu-Ni alloy	LSV	16–200	0.7	11	0.998	[4]
Porous copper nanoclusters	LSV	6.25–300	5.26	5	0.9993	[5]
		300–3500	3.53		0.9918	
Cu nanowire	LSV	10–400	0.7143	3.0	0.998	[6]
Cu on Pt	Amperometry	100–4000	3.931	–	0.9951	[7]
Cu nanoparticles on multiwall carbon nanotubes reduced graphene oxide	SWV	0.1–75	0.2257	0.02	0.9992	[8]
This work	Amperometry	50–600	5.647	12.2	0.9974	

a The diameter and length of extruded Cu wire were used to calculate the surface of our nanostructured electrode for comparison.

**Table 2:** Stability performance of the Cu-NWs electrochemical sensor.

Testing cycle	*I*_m_	RDS (%)
Day 1	− 1.823	2.08
Day 3	− 1.741	0.56
Day 14	− 1.859	5.58
Overall	–	6.08

The XRD patterns of the Cu wires in Fig. 1 show the crystal structural changes at each processing stage. After thermal oxidation, diffraction peaks assigned to CuO and Cu_2_O can be observed and the majority of the peaks belong to Cu_2_O crystal. After electrochemical (EC) reduction, the metallic Cu peaks are well recovered with negligible oxides peaks. Compared to the pristine Cu wires, the diffraction peaks of Cu wires after electrochemical reduction are broader.

Fig. 2 shows the CV diagram of Cu electrodes with and without surface nanostructures in N_2_ purged NaOH (50 mM) electrolyte. The oxidation peak of nanostructured Cu wire improved enormously compared with the bare one in the voltage range from − 0.4 V to − 0.2 V. The charge of Cu_2_O formation in bare and nanostructured Cu wire electrodes, calculated by integrating the oxidation peak area, are 0.266 mC and 17.258 mC, respectively. By assuming the required charge quantity to form a monolayer Cu_2_O is 180 μC cm^−2^ [1], the ESA of pristine Cu wire and Cu-NWs is 1.478 cm^2^ and 95.88 cm^2^, respectively.

In Fig. 3(a), no obvious reduction peak is observed over pristine Cu wires electrode in the presence of nitrate. The well-defined reduction peaks of nitrate are found in the potential range of − 0.4 to − 0.5 V for wires after electrochemical reduction (Cu-NWs). What's more, the peak current rose gradually with the increase of nitrate concentration.

Fig. 4 shows the dependence of cathodic peak current on scan rate (*v*). LSV curves were measured with scan rate in the range from 10.0 to 100.0 mV s^−1^. The peak current due to reduction of nitrate displayed a linear relationship with the square root of scan rate.

Fig. 5 shows the anti-interference performance of the surface nanostructured Cu wire. With sequential addition of different interference anions of ${\mathrm{Cl}}^{-}$, $H_{2}{\mathrm{PO}}_{4}^{-}$ or ${\mathrm{HCO}}_{3}^{-}$, the response currents almost remain unchanged compared with the blank electrolyte. However, a current increase was found when 1 mM ${\mathrm{NO}}_{2}^{-}$ anion was present in the solution.

Table 2 displays the stability of nanowire Cu as electrochemical sensor for nitrate detection. The measurements were conducted in a solution containing 200 μM nitrate at − 0.46 V (vs. SCE), where *I*_m_ represents the average current for two tests (time interval 15 min) and RDS the relative standard deviation for the successive measurements. No obvious change was observed when repeating the test within 24 h, with RDS less than 5.58%. For long term stability, the *I*_m_ exhibits fluctuation shape with 6.08% (RDS) in 14 days.

## 2 Experimental design, materials and methods

### 2.1 Preparation of Cu nanowires electrode

Cu wires of 0.2 mm in diameter were prepared by wire-drawing under room temperature using pure copper. In a typical preparation, Cu wires were sonicated in 1 M HCl solution for 3 min and then put into deionized water for 3 min to remove surface oxide impurities. The Cu wires were annealed in air at 600 °C for 4 h with a heating rate of 10 °C/min. The metallic copper nanowire arrays were then obtained using electrochemical reduction method at − 0.4 V (vs. RHE) in 0.1 M KOH solution purged with N_2_ gas. Copper oxide nanowires were completely reduced when the cathodic current reached a stable, near-zero horizontal.

### 2.2 Electrochemical measurement

Electrochemical measurements were conducted on a Zahner potentiostat in a three-electrode configuration, with platinum net as counter electrode and SCE as the reference electrode. The electrolyte was purged with N_2_ gas before measurement. The as-prepared Cu wires were cut into 5 cm for the measurement. Electrocatalytic active surface area (ESA) of the work electrode was measured through cyclic voltammetry (CV) in a 50 mM NaOH electrolyte at 5 mV s^−^^1^. Linear sweep voltammetry (LSV) was employed to characterize the ability of electrodes for nitrate reduction at 40 mV s^−^^1^. Amperometry (IT) measured at a constant − 0.46 V (vs. SCE) was used to detect the concentration of nitrate in water. Both of the LSV and IT were carried out in a 0.1 M Na_2_SO_4_ electrolyte at pH = 2.
