# Supplementary material for: Structure and electrochemical properties of copper wires with seamless 1D nanostructures
Source: Data Brief. 2018 Feb 3;17:747–52. doi: 10.1016/j.dib.2018.01.097 (PMC5988511; doi:10.1016/j.dib.2018.01.097)
Supplement: Supplementary file 1 — Supplementary material [file mmc1.docx]

Conflict of Interest

No competing financial interests.
